# Supplementary figures and images for: Endometrial Cancer in Pre-Menopausal Women and Younger: Risk Factors and Outcome
Source: Int J Environ Res Public Health. 2022 Jul 25;19(15):9059. doi: 10.3390/ijerph19159059 (PMC9330568; doi:10.3390/ijerph19159059)

Flow Chart for Study Design

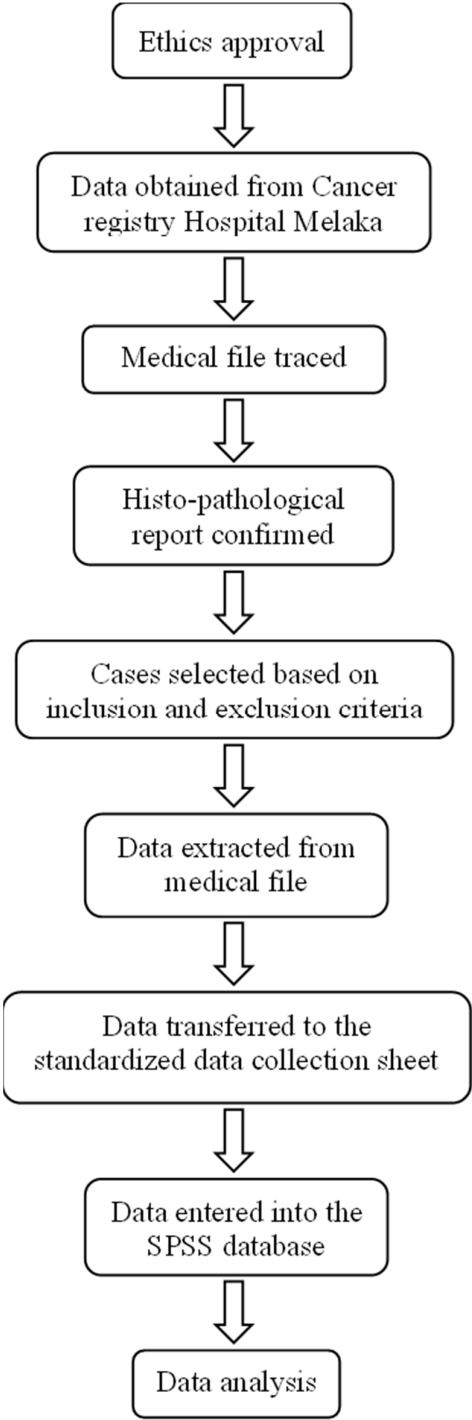

Supplement: Supplementary file 1 [file ijerph-19-09059-s001.zip › ijerph-1723117-supplementary.pdf]
